# Evaluation of retinal nerve fiber layer thickness measurements using optical coherence tomography in patients with tobacco-alcohol-induced toxic optic neuropathy

**DOI:** 10.4103/0301-4738.71694

**Published:** 2010

**Authors:** Suresh Ramchandani, Sushama Ramchandani, Indrajyotkaur Ahuja

**Affiliations:** Shivam Eye Foundation, Nerul (E), Navi Mumbai, Maharashtra, India

Dear Editor,

We read with great interest the article by Moura *et al*., titled ’Evaluation of retinal nerve fiber layer thickness measurements using optical coherence tomography in patients with tobacco-alcohol-induced toxic optic neuropathy’.[1] There are a few points we would like to point out to make the article more pertinent.

The authors have recorded and analyzed optical coherence tomogram (OCT) findings in cases of established toxic neuritis due to alcohol and tobacco. What was the purpose of doing nerve fiber analysis? Was it for academic purposes to document changes in nerve fiber layer or was it for diagnosis of the disease? (Which was already made).

Toxic-nutritional optic neuropathies are characterized by selective maculo-papillar bundle involvement, leading to central or caecocentral scotomas.[2]

Diagnosis of the condition is made by careful history, bilateral nature and slow onset.[2] Investigations required to rule out other causes are usually visual field examination and visual evoked potentials (VEP).

Multifocal electroretinogram (ERG) or OCT are tests done more to document whether the disease is affecting only the optic nerve or the nerve fiber layer as well.[3] Although the clinical findings seen in tobacco-alcohol amblyopia can occur in any disease of anterior visual pathway from the retina to the optic tract, and there is little evidence to suggest that the locus of pathology is restricted to the optic nerve,[3] these tests are usually not required in clinical practice but can be used as academic tools.

Any test to be really conclusive should have high sensitivity and specificity. In toxic neuropathy, OCT is neither sensitive (as shown in Case 3) nor specific (as thinning of retinal nerve fiber layer can occur in several conditions).

The authors have not mentioned whether they carried out a VEP in any of their patients. VEP is a sensitive test for optic nerve disorders though it is not specific for any particular disease.

In the third case there was thickening of the nerve fiber – inferiorly in the right eye and superiorly in the left eye suggesting edema,[1] this should have given rise to corresponding superior and inferior arcuate scotomas rather than the central scotoma. This again underlines the non-sensitive and non-specific nature of the procedure.

To summarize, OCT is an excellent tool, but its use in toxic neuropathies should be academic rather than clinical as better tests like perimetry and VEP are already available.
